# Rare skin manifestation of tularemia: Sweet syndrome

**DOI:** 10.1590/0037-8682-0242-2024

**Published:** 2025-01-27

**Authors:** Ömer Karaşahin

**Affiliations:** 1 Erzurum Research and Education Hospital, Department of Infectious Diseases and Clinical Microbiology, Erzurum, Turkey.

**FIGURE 1:**
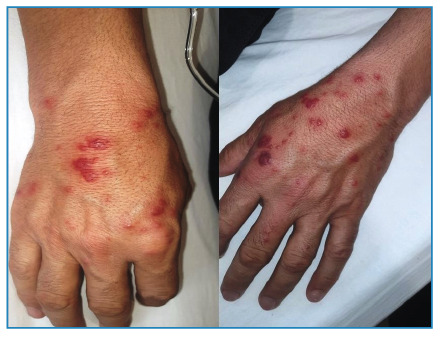
Erythematous plaques on the dorsal surface of both hands.

**FIGURE 2:**
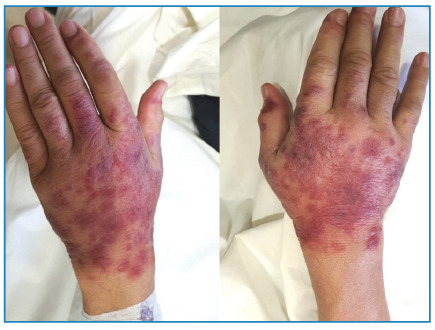
Worsening of hand lesions.

**FIGURE 3:**
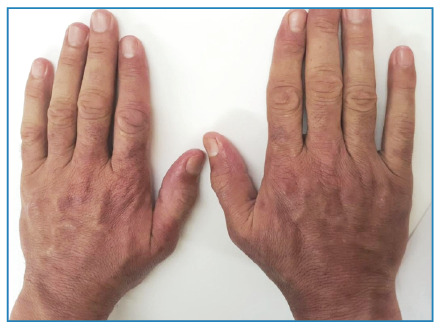
Lesion resolution.

A 49-year-old man was admitted with a 10-d history of fever, chills, and myalgia along with a 2-d history of painful skin rash on the hands. Physical examination revealed asymmetrical erythematous plaques on the dorsal surfaces of both hands (Figure 1). Additionally, a lymphadenopathy measuring 2.5-3.0 cm in size was noted in the left cervical region. Laboratory tests showed elevated C-reactive protein levels and neutrophilic leukocytosis. Ultrasonography revealed a cystic-necrotic lymph node in the left cervical region. A subsequent *Francisella tularensis* microagglutination test yielded a positive result, with a titer of 1:640, and streptomycin treatment (10 mg/kg twice daily) was initiated. Histopathological examination of the skin lesions revealed edema, hemorrhage, and inflammation, including polymorphonuclear leukocytes in the papillary dermis, which is consistent with Sweet syndrome. On the 5^th^ d of hospitalization, the size and pain intensity of hand lesions increased (Figure 2). Methylprednisolone was added at a dose of 40 mg/day and discontinued after 7 d. After 14 d of antibiotic therapy, the patient’s clinical symptoms completely resolved, and the laboratory results improved (Figure 3). Sweet syndrome, believed to be a hypersensitivity reaction to bacterial, viral, or tumor antigens, was initially reported to be associated with tularemia by Ruiz et al.^1^. The typical cutaneous manifestations of tularemia include papular rash, erythema nodosum, and erythema multiforme^1^ ^,^ ^2^. Our patient presented with a rare cutaneous manifestation of tularemia. Furthermore, unlike the two previously reported patients, our patient showed no clinical response to antibiotic treatment alone^1^ ^,^ ^3^. Therefore, methylprednisolone was added to his treatment.
